# Supplementary material for: Prediction of suicidal ideation risk in a prospective cohort study of medical interns
Source: PLoS One. 2021 Dec 2;16(12):e0260620. doi: 10.1371/journal.pone.0260620 (PMC8639060; doi:10.1371/journal.pone.0260620)
Supplement: S1 Fig — Notes: Risk curves calculated for 2015 cohort test set by applying prediction models of suicidal ideation during internship constructed from 2012–2014 cohorts training set. For each risk curve, observations are ordered from highest risk of SI during internship to lowest risk of SI during internship. The rug plot underneath each risk curve indicates observations with suicidal ideation. SI = suicidal ideation; BASE = Model includes baseline predictors of suicidal ideation; BASE+PRIOR = Model includes base + prior quarter predictors of suicidal ideation; BASE+PRIOR+CUR = Model includes base+prior+current quarter predictors of suicidal ideation; No SI Covariates = Model includes base+prior+current quarter predictors of suicidal ideation except for baseline and prior quarter suicidal ideation. (PDF) [file pone.0260620.s001.pdf]

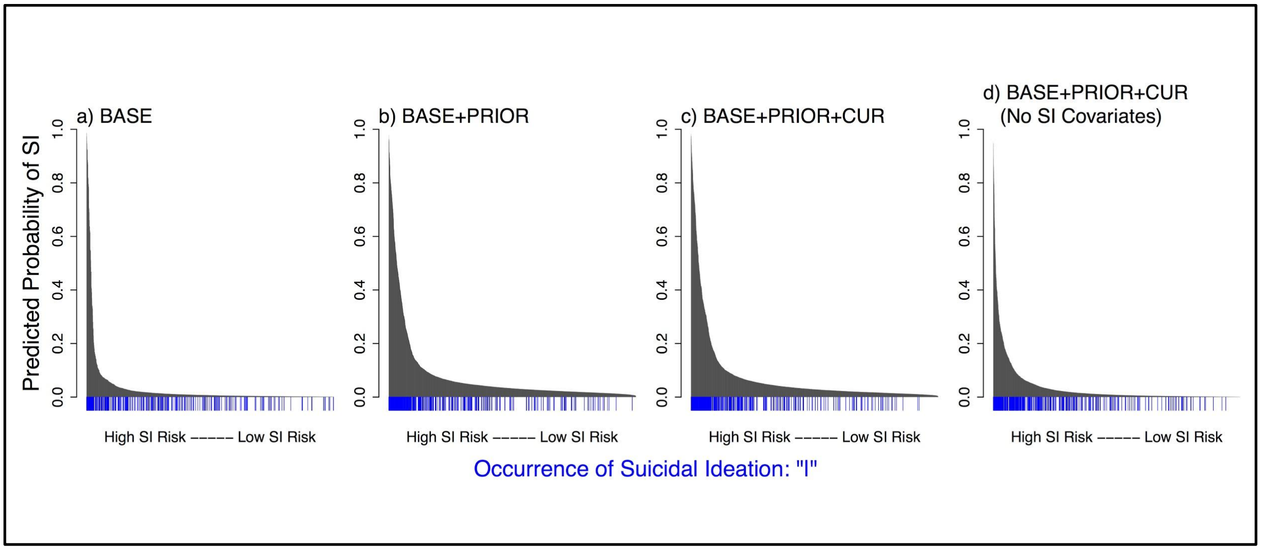

S1 Fig. Risk of Suicidal Ideation During Internship Across All Observations by Prediction Model, 2015 Cohort Test Set

Notes: Risk curves calculated for 2015 cohort test set by applying prediction models of suicidal ideation during internship constructed from 2012-2014 cohorts training set. For each risk curve, observations are ordered from highest risk of SI during internship to lowest risk of SI during internship. The rug plot underneath each risk curve indicates observations with suicidal ideation. SI = suicidal ideation; BASE = Model includes baseline predictors of suicidal ideation; BASE+PRIOR = Model includes base + prior quarter predictors of suicidal ideation; BASE+PRIOR+CUR = Model includes base+prior+current quarter predictors of suicidal ideation; No SI Covariates = Model includes base+prior+current quarter predictors of suicidal ideation except for baseline and prior quarter suicidal ideation.
