# Supplementary material for: Prediction of suicidal ideation risk in a prospective cohort study of medical interns
Source: PLoS One. 2021 Dec 2;16(12):e0260620. doi: 10.1371/journal.pone.0260620 (PMC8639060; doi:10.1371/journal.pone.0260620)
Supplement: S1 Table — Notes: Participants included in the above table were incoming first-year resident physicians (interns) that were assessed through the prospective cohort Intern Health Study. All interns in the table had complete baseline data and data for all tested explanatory variables over one set of consecutive internship quarter-intervals. Intern characteristics were self-reported. SI = suicidal ideation; SD = standard deviation. aNo reported SI during internship. bNumber of unique subjects in the given data set. cp-value for Pearson’s chi-squared test of independence. dAssessed via the NEO-Five Factor Inventory. ep-value for the Satterthwaite two-sample t-test. fAssessed via the Patient Health Questionnaire-8. gAssessed via the General Anxiety Disorder-7. (PDF) [file pone.0260620.s002.pdf]

S1 Table. Descriptive Univariable Analysis of Suicidal Ideation During Internship and its Association With Intern Demographics and Baseline Mental Health, 2012-2014 Cohorts Training Set

	SI During Internship	No SI During Internship[a]	*p*
**Number of Interns**[b]	367	1,926	
Baseline Characteristics			
**Suicidal Ideation**, *n* (%)	59 (16.1%)	13 (0.7%)	3.2×10^{-54} [c]
**Neuroticism Score**,[d] Mean (SD)	26.5 (8.6)	19.8 (8.1)	1.5×10^{-44} [e]
**Depressive Symptoms Score**,[f] Mean (SD)	4.3 (3.9)	2.1 (2.4)	4.6×10^{-23} [e]
**Anxiety Score**,[g] Mean (SD)	4.0 (3.7)	2.4 (2.9)	8.1×10^{-15} [e]
**Personal History of Depression**, *n* (%)	228 (62.1%)	799 (41.4%)	3.2×10^{-13} [c]
**Male Sex**, *n* (%)	199 (54.2%)	943 (49.0%)	0.1 [c]

Notes: Participants included in the above table were incoming first-year resident physicians (interns) that were assessed through the prospective cohort Intern Health Study. All interns in the table had complete baseline data and data for all tested explanatory variables over one set of consecutive internship quarter-intervals. Intern characteristics were self-reported. SI = suicidal ideation; SD = standard deviation.

[a]No reported SI during internship.

[b]Number of unique subjects in the given data set.

[c]*p*-value for Pearson's chi-squared test of independence.

[d]Assessed via the NEO-Five Factor Inventory.

[e]*p*-value for the Satterthwaite two-sample *t*-test.

[f]Assessed via the Patient Health Questionnaire-8.

[g]Assessed via the General Anxiety Disorder-7.
