# Supplementary material for: Prediction of suicidal ideation risk in a prospective cohort study of medical interns
Source: PLoS One. 2021 Dec 2;16(12):e0260620. doi: 10.1371/journal.pone.0260620 (PMC8639060; doi:10.1371/journal.pone.0260620)
Supplement: Attachment — Submitted filename: Response to Reviewers.docx [file pone.0260620.s003.docx]

Thank you for the helpful and constructive feedback. While revising the manuscript, we made a dedicated effort to fully address each comment.

We have replied to the Academic Editor and reviewer comments below. Please note that the text of the Academic Editor and reviewer comments is below in bold. Our responses follow in normal font.

**Academic Editor (Comments to the Authors):**

1. **I would echo the need for additional consideration of risk factors in the discussion.**

Upon further reflection, we agree with the above comment and have made substantial revisions to the Discussion section of our manuscript. The revised section provides further discussion of significant risk factors identified during our analysis (e.g., previous suicidal ideation, medical errors, work hours, sleep hours, neuroticism, depression, sex). (p. 15-17)

2. **Additionally, the manuscript includes very little literature review in the introduction.**

To address the above comment, we expanded the Introduction section of the manuscript to provide a more thorough literature review. In particular, the revised Introduction section reviews literature on Joiner’s Interpersonal Theory of Suicide, including empirical tests of the Interpersonal Theory’s hypotheses. (p. 2-3)

3. **The specific risk factors that were measured are not justified …**

We agree with the above comment. To wit, we added two paragraphs to the “Survey data” section of the manuscript that provide a rationale for the specific risk factors that were measured in our study. (p. 4-5)

4. **[T]here is no consideration of theories of suicide risk, such as Joiner's interpersonal theory or other recent ideation-to-action frameworks. Inclusion of these, and interpretation of results in this context, would greatly strengthen the paper.**

As mentioned in the response to comment #2, we revised the manuscript to incorporate Joiner’s Interpersonal Theory of Suicide Risk. In addition to the literature review mentioned above, we used Joiner’s Interpersonal Theory in the Discussion section of our revised manuscript to provide added context to our findings.

5. **Please ensure that your manuscript meets PLOS ONE's style requirements, including those for file naming. The PLOS ONE style templates can be found at**
   [**https://journals.plos.org/plosone/s/file?id=wjVg/PLOSOne_formatting_sample_main_body.pdf**](https://journals.plos.org/plosone/s/file?id=wjVg/PLOSOne_formatting_sample_main_body.pdf) **and** 
   [**https://journals.plos.org/plosone/s/file?id=ba62/PLOSOne_formatting_sample_title_authors_affiliations.pdf**](https://journals.plos.org/plosone/s/file?id=ba62/PLOSOne_formatting_sample_title_authors_affiliations.pdf)

We apologize for several formatting oversights in the previous version of the manuscript. We believe the revised manuscript now adheres to the journal’s style requirements. Specifically, we made the following formatting changes:

- We added page numbers.
- We changed the abstract from a structured abstract to an unstructured abstract.
- We removed the page break between the Abstract and Introduction sections.
- We moved Table 1 and Table 2 so that they are included directly after the respective paragraph in which each table is first cited.
- We removed the page break between the Conclusions and Acknowledgements sections as well as the page break between the Acknowledgements and References sections.
- We revised the file names to match the journal’s suggested style.

**Reviewer #1 (Comments to the Authors):**

1. **I think that a theory explaining the risk of suicide on medical students, interns, résidents... is lacking
   (see the interpersonal theory on suicide and the work on physicians or medicla students)
   cite and discuss (Cornette et al 2009; Fink-Miller 2015, Loas et al PLOS, 2018, 2019).**

We agree with this comment. As stated in our responses to comments #2 and #4 from the Academic Editor, we revised the manuscript to incorporate Joiner’s Interpersonal Theory of Suicide Risk. We also added citations for Cornette et al. (2009), Fink-Miller (2015), Loas et al. (2018), and Loas et al. (2019) and discuss these studies in the Introduction section of our revised manuscript (p. 2-3). We believe the discussion of these studies adds valuable context to our research.

**Reviewer #2 (Comments to the Authors):**

1. **This is an important and well written paper that presents the overall results well in the abstract, but, oddly, does not include any substantive discussion about the risk factors identified in the training set (high neuroticism, personal history of depression etc) in the discussion, apart from the male demographic discussion. These merit at least a detailed paragraph of discussion - what is the potential biological significance/vulnerability of a past history of depression and high neuroticism and could these be screened for in addition to using tools like the phq9? Can such fairly easily identified risk factors be used by training programs?**

We agree. As described in our responses to comments #1 and #4 from the Academic Editor, we made substantial revisions to the Discussion section of our manuscript. The revised section provides further discussion of significant risk factors identified during our analysis (e.g., previous suicidal ideation, medical errors, work hours, sleep hours, neuroticism, depression, sex). (p. 15-17)

2. **The authors also mention "second victim syndrome" in the discussion with reference to long hours of work and medical errors - but there is an increasing literature describing the impact of trauma on residents (especially repeat trauma) which could explain some of the longitudinal results presented, and a recent paper (Yellowlees et al) also described a link between Adverse Childhood Experiences (ACE'S) and burnout, which might be a more specific background reason and vulnerability than "neuroticism" as measured in this study.**

To address the above comment, we revised the Discussion section of our manuscript. The revised Discussion section describes the possible effect of trauma (including adverse childhood experiences) on medical resident burnout and suicidal ideation. (p. 15-16)
