# Supplementary material for: Prediction of suicidal ideation risk in a prospective cohort study of medical interns
Source: PLoS One. 2021 Dec 2;16(12):e0260620. doi: 10.1371/journal.pone.0260620 (PMC8639060; doi:10.1371/journal.pone.0260620)
Supplement: Attachment — Submitted filename: Response to Reviewers.docx [file pone.0260620.s004.docx]

Thank you again for the helpful and constructive feedback. While revising the manuscript, we made a dedicated effort to fully address each comment.

We have replied to the Academic Editor comments below. Please note that the text of the Academic Editor comments is below in bold. Our responses follow in normal font.

**Academic Editor (Comments to the Authors):**

1. **There a few minor concerns remaining. One is that while this is an observational design the manuscript includes some causal language. Please revise these to clarify what can be concluded.**

**[Example] 1. abstract, line 43: “…increased the risk of suicidal ideation…”**

**[Example] 2. abstract, line 48: “…leading to more medical errors…”**

**[Example] 3. page 14, line 335: “… that increased the…”**

We agree that our results should be interpreted cautiously given the observational study design. As such, we state the following in the Limitations section of our manuscript:

“As with any observational study, we do not know if the factors we identified as being associated with increased risk of suicidal ideation are causal for suicidal ideation, or if they are the result of other unmeasured causal factors…” (p. 17)

To further address the above comments, we carefully reviewed the manuscript to identify and revise remaining causal language (including the phrases noted above by the Academic Editor). Please see the “Track Changes” version of the manuscript for specific edits.

2. **[I]n the header for Table 2, please clarify the timing of the SI outcome.**

We revised the header for Table 2 to clarify that the outcome variable was suicidal ideation during the current quarter of internship. The full title is given below.

“Table 2. Logistic Mixed Effects Multiple Regression Analysis Predicting Current Quarter Suicidal Ideation from Intern Mental Health, Demographics, and Internship Characteristics” (p. 11)

3. **Finally, statistical significance is binary rather than continuous. On page 11, line 249, please revise either to indicate that these were the significant predictors or the strongest predictors based on effect size (OR), rather than the “most significant” predictors.**

Thank you for the above comment. We revised several sentences of the fourth Results paragraph to incorporate this feedback. The edited sentences are provided below.

“We found that prior quarter suicidal ideation (Odds Ratio (OR) = 7.84, *p* = 4.4 x 10^−32^, baseline suicidal ideation (OR = 5.41, *p* = 2.5 x 10^−11^), increase in self-reported work hours from the previous quarter (OR = 1.34, *p* = 4.0 x 10^−6^), current quarter self-reported medical errors (OR = 1.80, *p* = 1.1 x 10^−5^), prior quarter depressive symptoms score (OR = 1.36, *p* = 1.8 x 10^−4^), baseline neuroticism score (OR = 1.33, *p* = 3.4 x 10^−4^), and baseline personal history of depression (OR = 1.45, *p* = 4.7 x 10^−3^) were significant predictors of current quarter suicidal ideation under a threshold of *p* < .01, holding all other model covariates constant. Table 2 shows additional predictors that were significant under less strict thresholds of *p* < .05 and *p* < .10 …” (p. 11)
